# Automated Pupillometry as an Assessment Tool for Intracranial Hemodynamics in Septic Patients

**DOI:** 10.3390/cells11142206

**Published:** 2022-07-15

**Authors:** Ilaria Alice Crippa, Paolo Pelosi, Armin Alvaro Quispe-Cornejo, Antonio Messina, Francesco Corradi, Fabio Silvio Taccone, Chiara Robba

**Affiliations:** 1 Department of Intensive Care, Erasme Hospital, Université Libre de Bruxelles, 1070 Brussels, Belgium; arminquispe@gmail.com (A.A.Q.-C.); fabio.taccone@ulb.be (F.S.T.); 2 Department of Anesthesiology and Intensive Care, San Marco Hospital, San Donato Group, 24040 Zingonia, Italy; 3 Department of Anesthesiology and Intensive Care, San Martino Policlinico Hospital, IRCCS for Oncology and Neurosciences, 16132 Genoa, Italy; paolo.pelosi@hsanmartino.it (P.P.); kiarobba@gmail.com (C.R.); 4 Department of Surgical Sciences and Integrated Diagnostics (DISC), University of Genoa, 16132 Genoa, Italy; 5 Humanitas Clinical and Research Center—IRCCS, 20089 Rozzano, Italy; antonio.messina@humanitas.it; 6 Department of Surgical Medical and Molecular Pathology and Critical Care Medicine, University of Pisa, 56126 Pisa, Italy; francesco.corradi@unipi.it

**Keywords:** cerebral autoregulation, cerebral perfusion pressure, intracranial pressure, pupillary light reflex, outcome, neurological pupil index, dilation velocity, constriction velocity, transient hyperaemic response test

## Abstract

Impaired cerebral autoregulation (CA) may increase the risk of brain hypoperfusion in septic patients. Sepsis dysregulates the autonomic nervous system (ANS), potentially affecting CA. ANS function can be assessed through the pupillary light reflex (PLR). The aim of this prospective, observational study was to investigate the association between CA and PLR in adult septic patients. Transcranial Doppler was used to assess CA and calculate estimated cerebral perfusion pressure (eCPP) and intracranial pressure (eICP). An automated pupillometer (AP) was used to record Neurological Pupil Index (NPi), constriction (CV) and dilation (DV) velocities. The primary outcome was the relationship between AP-derived variables with CA; the secondary outcome was the association between AP-derived variables with eCPP and/or eICP. Among 40 included patients, 21 (53%) had impaired CA, 22 (55%) had low eCPP (<60 mmHg) and 15 (38%) had high eICP (>16 mmHg). DV was lower in patients with impaired CA compared to others; DV predicted impaired CA with area under the curve, AUROC= 0.78 [95% Confidence Interval, CI 0.63–0.94]; DV < 2.2 mm/s had sensitivity 85% and specificity 69% for impaired CA. Patients with low eCPP or high eICP had lower NPi values than others. NPi was correlated with eCPP (r = 0.77, *p* < 0.01) and eICP (r = −0.87, *p* < 0.01). Automated pupillometry may play a role to assess brain hemodynamics in septic patients.

## 1. Introduction

Brain dysfunction during sepsis is frequent and it is associated with increased mortality and long-term cognitive impairment among survivors [1]. Brain hypoperfusion has been suggested to contribute to brain dysfunction in critically ill septic patients. Alterations in different physiological mechanisms aiming at the maintenance of adequate cerebral blood flow (CBF) are the main determinants of brain hypoperfusion [2,3].

Cerebral hemodynamics depends on the interaction of several mechanisms. The autonomic nervous system (ANS) controls small- and medium-sized vessel diameters through the sympathetic (SNS) and parasympathetic nervous system (PNS) [4,5,6,7]. Cerebral autoregulation (CA) relies on the reaction of arterioles to transmural pressure and is partially influenced by the ANS modulation of basal vasomotor tone and cerebral vasculature responses to hemodynamic challenges [7]. CA counteracts the variations in cerebral perfusion pressure (CPP) by regulating arteriolar diameter, preventing the occurrence of brain hypoperfusion in case of reduced CPP (i.e., by decreasing cerebrovascular resistance) or brain hyperaemia in case of hypertension (i.e., by increasing cerebrovascular resistance) [8]. The ranges of CPP within which CA is efficient can be variable among healthy individuals [9], and markedly reduced or shifted towards higher CPP values in case of acute brain injury [10] or critical illness [11,12]. ANS manipulation affects CA in healthy subjects [13], and its alteration is associated with worse cerebral autoregulation in patients with acute brain injury [14,15]. Furthermore, ANS dysfunction has been suggested to occur during sepsis [16,17]. Previous studies showed that CA is altered in patients with sepsis [8], thus potentially exposing patients to brain hypoperfusion also when currently recommended mean arterial pressure (MAP) values are met [18]. Transcranial Doppler (TCD) is a non-invasive, safe technique which allows bedside investigation of CA [19] and provides a non-invasive estimation of CPP and ICP [20,21,22]. TCD also requires a trained operator, an ultrasound machine and an off-line analysis of gathered data.

Pupillary light reflex (PLR) describes the constriction and subsequent dilation of the pupils in response to light in the result of the antagonistic actions of the iris sphincter—i.e., innervated by the parasympathetic nervous system (PSN)—and dilator muscles—i.e., sympathetic nervous systems (SNS) (14). As such, PLR represents the balance between PNS and SNS activation and could be used to assess ANS dysfunction [23]. PLR may be assessed by automated pupillometers. Automated pupillometers are portable devices, with a short learning curve and which does not require complex off-line analysis. A first study indicated no relationship between PLR-derived Neurological Pupil Index (NPi) and CA in critically ill patients [24], but other available variables from the automated pupillometry could potentially be useful to evaluate CA.

The aim of this study was to assess the correlation between automated pupillometry PLR-derived variables with the assessment of autoregulation in critically ill patients with sepsis. Furthermore, we evaluated whether the same variables were correlated with the non-invasive estimation of CPP and ICP.

## 2. Materials and Methods

### 2.1. Study Design and Population

This was a prospective observational study including adult (>18 years) consecutive patients admitted with the diagnosis of sepsis [25] in the Intensive Care Unit (ICU) of San Martino Policlinico Hospital, Genova from 1 September 2021 to 1 February 2022 who underwent transient hyperaemic response test (THRT) and pupillometer assessment as for clinical practice. Exclusion criteria were: acute or chronic intracranial disease (i.e., brain tumour, cerebrovascular disease, dementia, previous traumatic brain injury); use of mechanical cardiac support (i.e., veno-arterial extracorporeal membrane oxygenation, left ventricular assist device, intra-aortic balloon pump counter-pulsation); severe hypotension (i.e. MAP < 50 mmHg) and/or severe hypercapnia (partial pressure of carbon dioxide, PaCO_2_ > 60 mmHg) or hypocapnia (PaCO_2_ < 30 mmHg) at the time of CA assessment; pregnancy; cardiac arrhythmias; pre-existing ocular disease or surgery; absence of invasive arterial blood pressure monitoring; and contraindications to perform a THRT test (i.e., known intra- or extra-cranial vascular stenosis). The study was approved by the local ethics review board “Comitato Etico Regione Liguria” (protocol n. CER Liguria: 23/2020), and written consent was obtained from patients’ next of kin, as all patients were unconscious at the time of inclusion according to the local regulations. This study is reported according to the “Strengthening the Reporting of Observational Studies in Epidemiology (STROBE)” statement guidelines for observational cohort studies [26].

### 2.2. Data Collection

Demographic data, pre-existing comorbid diseases and the Acute Physiology and Chronic Health Evaluation (APACHE) II score on admission were recorded. Physiological variables, such as heart rate (HR), arterial pressure (AP), peripheral oxygen saturation (SpO_2_), and body temperature were collected from the local patient data monitoring system. Additional variables (i.e., spontaneous or mechanical ventilation parameters; inspired oxygen fraction, FiO_2_; ventilation mode; respiratory rate; positive end-expiratory pressure, PEEP; arterial blood gases; lactate concentrations; central venous oxygen saturation; ScvO_2_) were collected on the day of assessment. The site of infection, the pathogen (s) involved, the administration of sedatives and of cardio-active medications (i.e., norepinephrine and/or dobutamine), and the outcome at ICU discharge were also collected.

### 2.3. Transcranial Doppler and CA Assessment

CA and PLR assessment were performed within 48 hours from ICU admission, according to local clinical practice, the availability of the devices and the operators. All assessments were performed when the patient was in a steady state condition, avoiding any stimuli, modifications of vasoactive drugs, sedation, fluid boluses or ventilator settings during the procedures. A head elevated over the bed at 30–45 degrees was maintained throughout the examination. In our unit, a THRT is generally performed to assess CA in all septic patients with no contraindications to the test [22]. THRT were performed by trained operators with extensive experience in TCD. Right or left middle cerebral artery (MCA) was identified using standard criteria [27] and insonated with a 2-MHz TCD ultrasound probe (Philips Spar-Q). A compression of the ipsilateral common carotid artery (CCA) was applied and considered reliable if it resulted in a sudden decrease in FV; compression was released after three seconds of stable FV reduction (i.e., at least 30% from baseline) [28] and an additional six seconds of total compression time. In case of preserved CA, compression of the ipsilateral CCA determines the reduction in MCA blood pressure, which in turns drives the vasodilation in the vascular bed distal to the MCA; the subsequent release of the compression should result in a transient increase in cerebral blood flow and, thus in cerebral blood flow velocity (FV). The FV waveform before CCA compression was considered as “baseline”; the maximal FV after compression release within five heartbeats was therefore identified; an increase in systolic FV > 10% from baseline was considered to indicate an “intact” CA, while lower values were considered as “impaired” CA [28]. The estimated CPP (eCPP) and estimated ICP (eICP) were calculated for each patient using a previously validated formula [29], which was applied on baseline FV waveform and MAP values. Normal eCPP (*n* eCPP) was defined as eCPP ≥ 60 mmHg, low eCPP (*l* eCPP) was defined as <60 mmHg; normal eICP (*n* eICP) was defined as eICP ≤ 16 mmHg, while high eICP (*h* eICP) was defined as >16 mmHg, as previously described [30].

### 2.4. PLR Assessment

PLR was evaluated using an automated pupillometer (NPi^®^-200 pupillometer; Neuroptics, Laguna Hills, CA, USA), which applies a stimulus of fixed intensity and duration in order to provide measurement of pupil size and PLR. In particular, the device measures pupil size, constriction rate, constriction velocity and dilation velocity, as well as the Neurological Pupil Index (NPi), which is an integrated index of pupillary function, based on an algorithm developed by the industry, ranging from 0 to 5. An NPi ≥ 3 is considered to be within normal ranges. All measurements were performed in complete darkness, just before CA assessment. The measurement required less than 30 s for each eye, with at least 1 min between the examination of the two pupils to obtain full recovery of baseline pupil diameter. Values from both eyes from the individual patient were averaged.

### 2.5. Study Outcomes

The primary outcome was to evaluate the relationships between variables derived from the automated pupillometry (such as dilation velocity and NPi) and CA, estimated through THRT. A secondary outcome was to assess whether these variables were associated with eCPP and/or eICP.

### 2.6. Statistical Analysis

Statistical analysis was performed using IBM SPSS Statistics 27.0 (IBM, Armonk, NY, USA), Prism (GraphPad Software Inc. 9.0, San Diego, CA, USA) and SAS OnDemand for Academics software (SAS Institute Inc., Cary, NC, USA). Continuous variables were expressed as median [25th–75th percentiles]. Categorical variables were expressed as count (percent). Distribution of continuous variables was tested by Shapiro-Wilk test and inspection of Q-Q plots. Student’s *t* test, Mann-Whitney test, and Kruskal-Wallis H test with post-hoc analysis or Chi-square test of homogeneity were applied for comparisons between groups, as appropriate. Correlations were assessed using Pearson’s *r* or Spearman’s *R* coefficients, as appropriate. The discriminative ability of dilation velocity to predict impaired CA was evaluated using receiver operating characteristic (ROC) curves with the corresponding area under the curve (AUROC). Youden’s index was computed to assess the optimal cut-off of the dilation velocity for sensitivity and specificity to predict impaired CA. Moreover, NPi values were evaluated among different combinations of eCPP and eICP (group I = low eCPP and high eICP; group II = low eCPP and normal eICP; group III = normal eCPP, as only one patient with normal eCPP had also high eICP). All tests are two tailed and the statistical significance was set at the *p* < 0.05 level (two-sided).

## 3. Results

### 3.1. Study Population and CA

Fifty-two patients were screened, and 40 patients were included in the final analysis (12 patients were excluded: four with brain injury; six with severe hypotension, two without consent). Twenty-one (53%) patients had impaired CA; no main differences in demographics were observed between patients with impaired and intact CA. Overall ICU mortality was 18%. Characteristics of the study cohort are reported in Table 1, transcranial Doppler and automated pupillometry findings are reported in Table 2.

### 3.2. Automated Pupillometry, eCPP and eICP

Median NPi values were 4.3 [3.6–4.6]; all patients had NPi within normal ranges. Median constriction velocity was 1.7 [1.5–2.3] mm/s and median dilation velocity was 1.7 [1.2–2.8] mm/s. Median eCPP was 58 [50–67] mmHg and 22/40 (55%) patients had low eCPP; median eICP was 9 [4–24] mmHg and 15/40 (38%) patients had high eICP. Fourteen out of 40 (35%) patients had low eCPP and high eICP (group I); 8/40 (20%) patients had low eCPP and normal eICP (group II); 18/40 (45%) patients had normal eCPP and either high eICP (1/40, 2%) or normal eICP (17/40, 43%) (group III).

### 3.3. Primary Outcome

**Figure 1:**
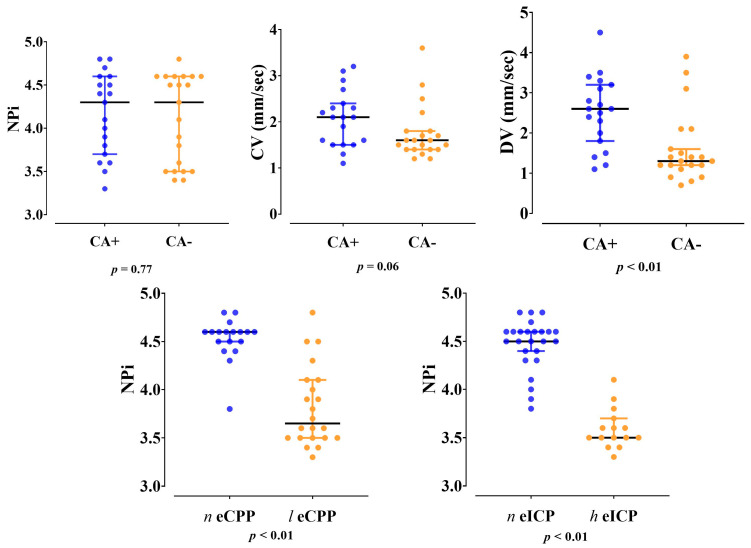
Differences in Neurological Pupil Index (NPi), constriction velocity (CV) and dilation velocity (DV) between patients with impaired (CA−) and intact (CA+) cerebral autoregulation; differences in Neurological Pupil Index (NPi) between patients with normal (*n* eCPP) and low (*l* eCPP) cerebral perfusion pressure or between those with normal (*n* eICP) and high (*h* eICP) intracranial pressure.

Dilation velocity was significantly lower in patients with impaired CA when compared to those with intact CA (1.3 [1.2–1.9] vs. 2.6 [1.8–3.2] mm/s; *p* < 0.01) (Figure 1). The AUROC for dilation velocity to predict impaired CA was 0.78 [95% CI 0.63–0.94], with a value of <2.2 mm/s having a sensitivity of 85 [95% CI 65–95] % and a specificity of 69 [95% CI 46–84] for impaired CA.

### 3.4. Secondary Outcome

**Figure 2:**
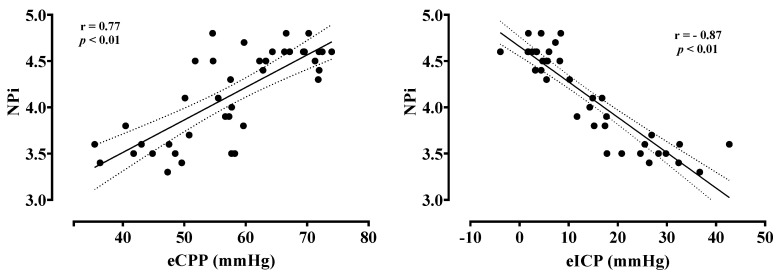
Correlation between estimated cerebral perfusion pressure (eCPP) and estimated intracranial pressure (eICP) with the Neurological Pupil Index (NPi).

**Figure 3:**
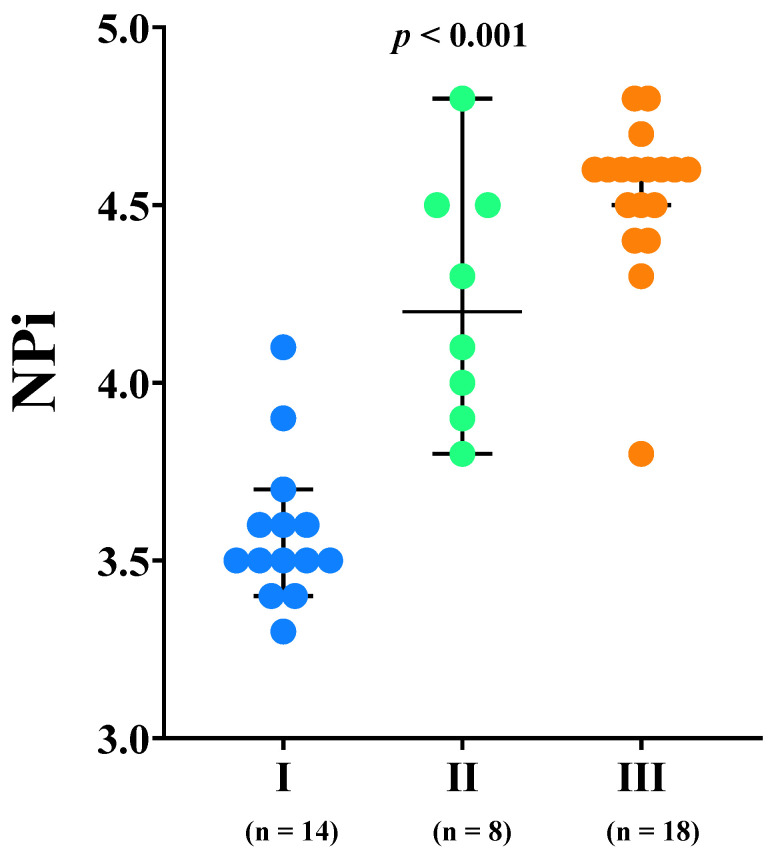
Neurological Pupil Index (NPi) values according to the different combination estimated cerebral perfusion pressure (eCPP) and estimated intracranial pressure (eICP): group I = low eCPP and high eICP; group II = low eCPP and normal eICP; group III = normal eCPP.

NPi values were lower in patients with low compared to normal eCPP values (3.7 [3.5–4.1] vs. 4.6 [4.5–4.6], *p* < 0.01, respectively) and in patients with high eICP compared to normal eICP values (3.5 [3.5–3.6] vs. 4.5 [4.3–4.6]; *p* < 0.01, respectively) (Figure 1). Patients with low eCPP had higher PaO_2_, lower PaCO_2_, lower lactate levels and lower arterial pH when compared to patients with normal eCPP (Supplementary Tables S1 and S2). Patients with high eICP were more likely to be female, to have higher MAP and higher PaO_2_ when compared to patients with normal eICP values. NPi was correlated with eCPP (r = 0.77, *p* < 0.01) and eICP (r = −0.87, *p* < 0.01) (Figure 2). No correlation between eCPP (r = 0.21; *p* = 0.19 and r = 0.13; *p* = 0.42, respectively) or eICP (r = −0.16; *p* = 0.32 and r = −0.10; *p* = 0.38 respectively) and constriction or dilation velocities was found. The values of NPi were significantly lower in patients with low eCPP and high eICP when compared to the other groups (*p* < 0.001; Figure 3).

## 4. Discussion

In the present study, we found that: (1) 53% of septic patients had impaired CA, 55% had low eCPP and 38% had high eICP; (2) DV at PLR evaluation was lower in patients with impaired CA than patients with intact CA; (3) all patients had normal pupillary function, as assessed by NPi. NPi was significantly correlated with eCPP and eICP; the lowest NPi values were observed among patients with low eCPP and high eICP. 

To the best of our knowledge, this is the first study exploring the role of pupillometry in the assessment of cerebral hemodynamics (CA, eCPP and eICP) in a homogeneous group of septic patients.

CA assessment holds an interest in critically ill septic patients, as cerebral perfusion optimization remains fundamental to avoid cerebral hypo/hyper-perfusion and secondary brain damage [1,8]. The alteration in CA may expose the patients to altered brain perfusion even at MAP levels that are within “normal ranges” according to current recommendations [31].

THRT is one of the most used methods to assess CA using TCD, and it relies on the modifications in intracranial flow velocities that occur after the ipsilateral CCA is briefly compressed and released [32]. THRT has been also validated against static measures of CA [22,33], it is minimally affected by anatomic vascular variations [34] and may be performed at the bedside without complications.

The neurogenic modulation of cerebral vasoreactivity and CA depends on ANS, which controls small- and medium-sized vessel diameters through the sympathetic (SNS) and parasympathetic nervous system (PNS) activity [4,5,6,7]. Although the exact underlying pathophysiological mechanism has not been determined yet, ANS dysfunction has been suggested to occur during sepsis and to involve excessive SNS activation and/or inappropriate PNS downregulation [16,17]. The vagus nerve projects to the locus coeruleus region of the brain, which controls pupil dilation both directly, through innervation of the pupillary dilator muscle, and indirectly, through modulation of PNS, which is the main determinant of pupil constriction [23]. Locus coeruleus, other than regulating PLR, has been shown to regulate arousal and to have an influence on cortical activity [35], which can be considerably altered in sepsis [36]. Considering the above-mentioned pathophysiological mechanisms occurring in sepsis, PLR assessment may be potentially helpful in the detection of ANS dysfunction and in the identification of septic patients at higher risk of CA failure. However, ANS dysfunction in sepsis involves complex mechanisms of action and feedback: receptor desensitization occurs in the case of excessive and prolonged ANS activation, but the exact timing, magnitude and regional differences of such desensitization is not known [16], thus further complicating the interpretation of possible signs of dysfunction.

Automated pupillometry (AP) has been applied in critical care settings to overcome the inter-operator poor agreement in conventional clinical assessment of pupil size and function [37]. While clinical PLR assessment can be influenced by sedation, analgesics and other physiological variables [38], AP has been developed to better quantify pupillary function and provides interesting prognostic information. Low NPi values have shown to be associated with increased ICP in traumatic brain injury patients [39], with midline shift in ischemic stroke patients [40] and after subarachnoid haemorrhage or at risk of herniation in patients with delayed cerebral ischemia [41,42]. Moreover, NPi ≤ 2 may identify patients with unfavourable outcomes after post-anoxic brain injury [43,44]. Few studies have evaluated PLR by the use of AP in septic patients [35,36]. In a cohort of 100 mixed medical-surgical patients on mechanical ventilation, reduced PLR and constriction velocity at day 3 were independently associated with the occurrence of delirium [45]. A large study on 214 critically ill patients, including 21% diagnosed with sepsis, confirmed the high accuracy of low DV in detecting an unreactive electroencephalography in non-anoxic critically ill patients [46]. Contrary to this, a previous study of 92 septic and non-septic patients did not show any correlations between NPi and CA [24]. In our cohort of septic patients, NPi did not differ between patients with intact and altered CA, while DV was shown to be the best pupillometry-derived variable to identify patients at risk of impaired CA. It is possible that, NPi being a composite index of total pupillary function, might be less sensitive than DV to alterations associated with sepsis.

Brain dysfunction during sepsis has a complex, multifactorial pathogenesis. Regional or global hypoperfusion is considered to be a main contributing factor [47]. Vasogenic edema, blood-brain barrier disruption or intracerebral vasodilation associated to protective ventilation strategies in the absence of an effective modulating mechanism may be responsible for increased ICP. In our cohort, 55% of septic patients had low eCPP and 38% had high eICP. A previous study on a smaller cohort found reductions in CPP < 60 mmHg in 73% and elevations in ICP > 15 mmHg in 47% of the 15 septic patients evaluated non-invasively through an algorithm providing CA status integration [48].

However, the non-invasive estimation of ICP has shown to be reliable in excluding elevated ICP, while the accuracy in the identification of patients with normal ICP was lower and the agreement between absolute values of invasively measured ICP and non-invasively estimated ICP was 33% [21]. Indeed, TCD measures blood flow velocity and not intravascular pressure. Blood flow velocity in intracranial arteries is affected to a different degree in its systolic, diastolic, and mean values by modifications in arteriolar diameter and cerebral vascular resistance, which is in turn determined by several mechanisms. As an example, a normal ICP with low MAP in the absence of reactive vasoconstriction due to cerebral autoregulation failure determines a greater reduction in the diastolic than in the mean velocity. The same effects on diastolic and mean flow velocities occur in case of increased ICP, despite a normal MAP [49]. In both cases, the ratio between diastolic and mean blood flow velocity decreases, leading to a correct estimation of low eCPP and an overestimation of ICP in the first case and a correct estimation of high eICP in the second case. Also, TCD explores the arteriolar bed. As such, it is less sensitive to changes in ICP caused by derangements in the CSF circulation or by increases in parenchyma volume—which would not be promptly transmitted to the arterial bed compared to the altered ICP of vasogenic origin [50]. However, such limitation does not apply to the high rate of low CPP found in our cohort. Such a result, together with results from previous studies indicating low eCPP [48], altered CA [8,18,51] and increased lower limit of CA [8] during sepsis might indicate that higher MAP values than currently recommended are beneficial in septic patients.

We also demonstrated that NPi is correlated with eCPP and eICP values, which is in line with previous studies on acute brain injury [39,40,52]. In particular, patients whose perfusion was compromised to a greater extent due to low eCPP together with high eICP showed the lowest value of NPi. Similarly, patients with normal eCPP and normal eICP (as only one patient with normal eCPP had high eICP) showed higher NPi values than patients with low eCPP and normal eICP. These findings might be related to a moderate increase in ICP or to insufficient CPP values. In fact, reduced CBF *per se* might also be responsible for very subtle pupillary abnormalities, identified with AP and NPi [52]. Also, as NPi showed great sensitivity in detecting elevated ICP in acutely brain injured patients, it is possible that increased ICP determines more pronounced or quicker alterations in ANS, which are detected by automated pupillometer PLR assessment.

Our study has some limitations that need to be acknowledged. This is a single-centre observational pilot study to explore potential applications of AP in critically ill septic patients. Therefore, we cannot draw any definitive conclusions on the causal relationship between ANS dysfunction, pupillary alterations, and impaired CA [16]. All patients underwent mechanical ventilation and thus were sedated to facilitate toleration to tracheal intubation and were mostly on vasopressors. Even though results are still conflicting [53,54,55,56,57,58,59], a recent study found that sedatives and vasopressors do not affect CA significantly in traumatic brain injury patients [60]. Other physiological variables, which may have an influence on CA (i.e., carbon dioxide and MAP), were in a near-physiological range in our cohort. Furthermore, given the short time required to perform a THRT, it is unlikely that the effects of sedation on cerebral flow-metabolism coupling and on cerebral vasculature basal tone has a detectable effect on CA as assessed by THRT. The same considerations are true for the potential effects of medications and physiological variables on PLR, which are still under debate [61,62,63,64]. However, we do acknowledge that CA was measured at a single time-point, and CA status might change over the course of sepsis in one individual patient. Similarly, eICP and eCPP were estimated at a single time point, early during the course of sepsis, thus not excluding the possibility of alterations later on during the ICU stay. FV modifications are proportional to CBF modifications only if the diameter of the insonated vessel does not change over time. When abrupt passive reduction in MAP is applied, a small-magnitude vessel vasodilation may occur [65,66]. The contribution of collateral perfusion through the circle of Willis is difficult to assess. Once the maximal dilation has occurred in vasculature distal to the compression point, the magnitude of the hyperaemic response is no longer proportional to the magnitude of the vasodilation achieved [34]. Finally, our study focused on pupil constriction and dilation, and other automated pupillometer-derived variables were not included in the analysis.

## 5. Conclusions

Pupillary dilation velocity was lower in critically ill septic patients with impaired CA compared to those with preserved CA. Lower NPi values were observed in patients with low eCPP and high eICP. Automated pupillometry may play a role in assessing brain hemodynamics in septic patients.

## Figures and Tables

**Table 1:** Characteristics of the study population in the overall population, in patients with intact and impaired cerebral autoregulation (CA).

	All (n = 40)	Intact CA (n = 19)	Impaired CA (n = 21)	*p*-Value
**Age, years**	74 [63–79]	76 [62–79]	67 [63–79]	0.77
**Male gender, n (%)**	23 (58)	10 (53)	13 (62)	0.75
**APACHE II score**	24 [19–29]	24 [19–29]	23 [19–26]	0.48
**Arterial hypertension, n (%)**	7 (18)	3 (16)	4 (19)	1.00
**Diabetes, n (%)**	5 (13)	2 (11)	3 (14)	1.00
**Coronary artery disease, n (%)**	2 (5)	1 (5)	1 (5)	1.00
**COPD, n (%)**	3 (8)	1 (5)	2 (10)	1.00
**CKD, n (%)**	1 (3)	0 (0)	1 (5)	1.00
**Obesity, n (%)**	13 (33)	4 (21)	9 (43)	0.19
**Smoking, n (%)**	4 (10)	3 (16)	1 (5)	0.33
**Source of infection:** - Abdomen - Lung - Genito-urinary tract - Blood	14 (35) 16 (40) 4 (10) 6 (15)	6 (32) 7 (37) 2 (11) 4 (21)	8 (38) 9 (43) 2 (10) 2 (10)	0.78
**ON THE DAY OF CA ASSESSMENT**				
**Heart rate, bpm**	88 [76–103]	89 [77–112]	85 [73–98]	0.22
**MAP, mmHg**	71 [66–76]	71 [67–77]	72 [66–75]	0.66
**Temperature, °C**	37.2 [36.9–37.6]	37.1 [36.9–37.4]	37.2 [37.1–37.7]	0.29
**Mechanical ventilation, n (%)**	36 (90)	17 (90)	19 (91)	1.00
**FiO_2_, %**	55 [50–60]	55 [50–60]	55 [48–60]	0.57
**PEEP, cmH_2_O**	8 [8–10]	8 [8–10]	10 [8–10]	0.36
**pH**	7.36 [7.35–7.39]	7.37 [7.35–7.41]	7.35 [7.34–7.38]	0.07
**PaO_2_, mmHg**	91 [69–101]	95 [74–101]	91 [64–103]	0.96
**PaCO_2_, mmHg**	41 [38–44]	41 [38–43]	41 [39–45]	0.35
**Lactate, mEq/L**	1.9 [1.5–2.3]	2.1 [1.8–2.3]	1.6 [1.1–2.6]	0.10
**Hb, g/dL**	9.1 [8.7–9.9]	9.2 [8.6–10.0]	9.1 [8.7–9.6]	0.75
**Cardio-active medications** - Norepinephrine, n (%) - Dobutamine, n (%) - Epinephrine, n (%)	38 (95) 8 (10) 7 (18)	18 (95) 4 (21) 3 (16)	20 (95) 4 (19) 4 (19)	0.76

APACHE acute physiology and chronic health evaluation; CKD chronic kidney disease; COPD chronic obstructive pulmonary disease; FiO_2_ fraction of inspired oxygen; FVm mean intracranial blood flow velocity, Hb haemoglobin; MAP mean systemic arterial pressure; PaCO_2_ arterial carbon dioxide partial pressure; PaO_2_ arterial oxygen partial pressure; PEEP positive end-expiratory pressure.

**Table 2:** Pupillometry and transcranial Doppler findings in the overall population in patients with intact and impaired cerebral autoregulation (CA).

	All (n = 40)	Intact CA (n = 19)	Impaired CA (n = 21)	*p*-Value
**FVm, cm/s**	55 [53–62]	54 [53–61]	59 [54–65]	0.60
**eCPP, mmHg**	58 [50–67]	58 [51–67]	58 [49–70]	0.83
**Low eCPP, n (%)**	22 (55)	10 (53)	12 (57)	1.00
**eICP, mmHg**	9 [4–24]	10 [4–21]	6 [3–25]	0.56
**High eICP, n (%)**	15 (38)	7 (37)	8 (38)	1.00
**NPi**	4.3 [3.6–4.6]	4.3 [3.7–4.6]	4.3 [3.5–4.6]	0.77
**Constriction velocity, mm/s**	1.7 [1.5–2.3]	2.1 [1.5–2.4]	1.6 [1.4–1.8]	0.06
**Dilation velocity, mm/s**	1.7 [1.2–2.8]	2.6 [1.8–3.2]	1.3 [1.2–1.9]	0.02
**Dead at ICU discharge, n (%)**	7 (18)	5 (26)	2 (10)	0.23

eCPP estimated cerebral perfusion pressure; eICP estimated intracranial pressure; FV blood flow in intracranial arteries; NPi neurological pupil index; ICU, intensive care unit.

## Data Availability

The data presented in this study are available on request from the corresponding author. The data are not publicly available due to ethical restriction.

## Supplementary Materials

The following supporting information can be downloaded at: https://www.mdpi.com/article/10.3390/cells11142206/s1, Method S1: STROBE Statement—Checklist of items that should be included in reports of cohort studies; Table S1: Characteristics of the cohort and the subgroup of patients with normal or low eCPP; Table S2: Characteristics of the cohort and the subgroup of patients with normal or high eICP.
