# Supplementary material for: Automated Pupillometry as an Assessment Tool for Intracranial Hemodynamics in Septic Patients
Source: Cells. 2022 Jul 15;11(14):2206. doi: 10.3390/cells11142206 (PMC9319569; doi:10.3390/cells11142206)
Supplement: Supplementary file 1 [file cells-11-02206-s001.zip › cells-1750455-supplementary.pdf]

Supplemental Methods S1. STROBE Statement—Checklist of items that should be included in reports of *cohort studies*.

	Item No	Recommendation	Page
**Title and abstract**	1	(*a*) Indicate the study's design with a commonly used term in the title or the abstract	1
		(*b*) Provide in the abstract an informative and balanced summary of what was done and what was found	1,2
**Introduction**			
Background/rationale	2	Explain the scientific background and rationale for the investigation being reported	3,4
Objectives	3	State specific objectives, including any prespecified hypotheses	4
**Methods**			
Study design	4	Present key elements of study design early in the paper	5
Setting	5	Describe the setting, locations, and relevant dates, including periods of recruitment, exposure, follow-up, and data collection	5
Participants	6	(*a*) Give the eligibility criteria, and the sources and methods of selection of participants. Describe methods of follow-up	5
		(*b*) For matched studies, give matching criteria and number of exposed and unexposed	NA
Variables	7	Clearly define all outcomes, exposures, predictors, potential confounders, and effect modifiers. Give diagnostic criteria, if applicable	6,5
Data sources/ measurement	8*	For each variable of interest, give sources of data and details of methods of assessment (measurement). Describe comparability of assessment methods if there is more than one group	*5,6*
Bias	9	Describe any efforts to address potential sources of bias	5,6
Study size	10	Explain how the study size was arrived at	NA
Quantitative variables	11	Explain how quantitative variables were handled in the analyses. If applicable, describe which groupings were chosen and why	7
Statistical methods	12	(*a*) Describe all statistical methods, including those used to control for confounding	7
		(*b*) Describe any methods used to examine subgroups and interactions	7
		(*c*) Explain how missing data were addressed	NA
		(*d*) If applicable, explain how loss to follow-up was addressed	NA
		(*e*) Describe any sensitivity analyses	NA
**Results**			

Participants	13*	(a) Report numbers of individuals at each stage of study—eg numbers potentially eligible, examined for eligibility, confirmed eligible, included in the study, completing follow-up, and analysed	8
		(b) Give reasons for non-participation at each stage	8
		(c) Consider use of a flow diagram	NA
Descriptive data	14*	(a) Give characteristics of study participants (eg demographic, clinical, social) and information on exposures and potential confounders	8, table 1
		(b) Indicate number of participants with missing data for each variable of interest	Table 1
		(c) Summarise follow-up time (eg, average and total amount)	NA
Outcome data	15*	Report numbers of outcome events or summary measures over time	8
Main results	16	(*a*) Give unadjusted estimates and, if applicable, confounder-adjusted estimates and their precision (eg, 95% confidence interval). Make clear which confounders were adjusted for and why they were included	8,9
		(*b*) Report category boundaries when continuous variables were categorized	7
		(*c*) If relevant, consider translating estimates of relative risk into absolute risk for a meaningful time period	NA
Other analyses	17	Report other analyses done—eg analyses of subgroups and interactions, and sensitivity analyses	7-9
**Discussion**			
Key results	18	Summarise key results with reference to study objectives	8,9
Limitations	19	Discuss limitations of the study, taking into account sources of potential bias or imprecision. Discuss both direction and magnitude of any potential bias	13
Interpretation	20	Give a cautious overall interpretation of results considering objectives, limitations, multiplicity of analyses, results from similar studies, and other relevant evidence	10-13
Generalisability	21	Discuss the generalisability (external validity) of the study results	10-13
**Other information**			
Funding	22	Give the source of funding and the role of the funders for the present study and, if applicable, for the original study on which the present article is based	NA

Supplemental Table S1. Characteristics of the cohort and the subgroup of patients with normal or low estimated cerebral perfusion pressure (eCPP).

	**All (n=40)**	**Normal eCPP (n=18, 45%)**	**Low eCPP (n=22, 55%)**	**p-value**
**Age, years**	74 [63-79]	67 [61-80]	76 [67-79]	0.48
**Male, n (%)**	23 (58)	11 (61)	12 (55)	0.76
**APACHE II score**	24 [19-29]	24 [18-30]	23 [19-26]	0.74
**Arterial hypertension, n (%)**	7 (18)	3 (16)	4 (19)	1.00
**Diabetes mellitus, n (%)**	5 (13)	3 (17)	2 (9)	0.64
**Coronary artery disease, n (%)**	2 (5)	0 (0)	2 (9)	0.49
**COPD, n (%)**	3 (8)	1 (6)	2 (9)	1.00
**CKD, n (%)**	1 (3)	1 (6)	0 (0)	0.45
**Obesity, n (%)**	13 (33)	7 (39)	6 (27)	0.51
**Tobacco abuse, n (%)**	4 (10)	1 (6)	3 (14)	0.61
**Source of infection:**				0.52
- **Abdomen**	14 (35)	7 (39)	7 (32)	
- **Lung**	16 (40)	3 (17)	1 (5)	
- **Genito-urinary tract**	4 (10)	6 (33)	10 (45)	
- **Blood**	6 (15)	2 (11)	4 (18)	
**ON THE DAY OF CA ASSESSMENT**				
**Heart rate, bpm**	88 [76-103]	89 [77-102]	87 [73-105]	0.90
**MAP, mmHg**	71 [66-76]	72 [68-76]	71 [63-78]	0.64
**Temperature, °C**	37.2 [36.9-37.6]	37.2 [36.7-37.5]	37.2 [37.0-37.7]	0.32
**Mechanical ventilation, n (%)**	36 (90)	17 (94)	19 (86)	0.61
**FiO_2, %**	55 [50-60]	55 [49-56]	55 [50-60]	0.72
**PEEP, cmH_2O**	8 [8-10]	8 [8-10]	8 [8-10]	0.70
**pH**	7.36 [7.35-7.39]	7.35 [7.34-7.38]	7.37 [7.35-7.41]	0.048
**PaO_2, mmHg**	91 [69-101]	84 [64-97]	98 [87-110]	0.03
**$PaCO_2$, mmHg**	41 [38-44]	43 [41-45]	40 [38-41]	<0.01
**Lactate, mEq/L**	1.9 [1.5-2.3]	2.1 [1.8-2.9]	1.6 [1.2-2.2]	<0.01
**Hb, gr/dL**	9.1 [8.7-9.9]	9.1 [8.7-9.8]	9.1 [8.7-9.9]	0.69

**Cardio-active medications**				
- **Norepinephrine, n (%)**	38 (95)	17 (94)	21 (96)	1.00
- **Dobutamine, n (%)**	8 (10)	3 (17)	5 (23)	0.71
- **Epinephrine, n (%)**	7 (18)	3 (17)	4 (18)	1.00
**TRANSCRANIAL DOPPLER AND PUPILLOMETRY FINDINGS**				
**FVm, cm/sec**	55 [53-62]	61 [54-65]	54 [48-56]	<0.01
**eCPP, mmHg**	58 [50-67]	68 [63-72]	50 [44-57]	<0.01
**eICP, mmHg**	9 [4-24]	4 [2-6]	19 [14-29]	<0.01
**Impaired CA, n (%)**	21 (53)	9 (50)	12 (55)	1.00
**NPi**	4.3 [3.6-4.6]	4.6 [4.5-4.6]	3.7 [3.5-4.1]	<0.01
**Constriction velocity, mm/sec**	1.7 [1.5-2.3]	2.0 [1.6-2.4]	1.6 [1.4-2.1]	0.13
**Dilation velocity, mm/sec**	1.7 [1.2-2.8]	2.1 [1.3-2.9]	1.5 [1.2-2.9]	0.23
**Dead at ICU discharge, n (%)**	7 (18)	3 (17)	4 (18)	1.00

APACHE II acute physiology and chronic health evaluation II, CAR cerebral autoregulation, CKD chronic kidney disease, COPD chronic obstructive pulmonary disease, eCPP estimated cerebral perfusion pressure, eICP estimated intracranial pressure, FiO2 fraction of inspired oxygen, FVm mean intracranial blood flow velocity, Hb haemoglobin, MAP mean systemic arterial pressure, NPi neurological pupil index, $PaCO_2$ arterial carbon dioxide partial pressure, PaO_2 arterial oxygen partial pressure, PEEP positive end-expiratory pressure.

Supplemental Table S2. Characteristics of the cohort and the subgroup of patients with normal or high estimated intracranial pressure (eICP).

	**All (n=40)**	**Normal eICP (n=25, 62%)**	**High eICP (n=15, 38%)**	**p-value**
**Age, years**	74 [63-79]	69 [63-79]	76 [66-80]	0.70
**Male, n (%)**	23 (58)	19 (76)	4 (27)	<0.01
**APACHE II score**	24 [19-29]	24 [18-29]	23 [22-26]	0.68
**Arterial hypertension, n (%)**	7 (18)	4 (16)	3 (20)	1.00
**Diabetes mellitus, n (%)**	5 (13)	3 (12)	2 (13)	1.00
**Coronary artery disease, n (%)**	2 (5)	0 (0)	2 (13)	0.14
**COPD, n (%)**	3 (8)	2 (8)	1 (7)	1.00
**CKD, n (%)**	1 (3)	1 (4)	0 (0)	1.00
**Obesity, n (%)**	13 (33)	7 (28)	6 (40)	0.50
**Tobacco abuse, n (%)**	4 (10)	3 (12)	1 (7)	1.00
**Source of infection:**				0.79
- **Abdomen**	14 (35)	10 (40)	4 (27)	
- **Lung**	16 (40)	2 (8)	2 (13)	
- **Genito-urinary tract**	4 (10)	9 (36)	7 (47)	
- **Blood**	6 (15)	4 (16)	2 (13)	
**ON THE DAY OF CA ASSESSMENT**				
**Heart rate, bpm**	88 [76-103]	88 [77-103]	85 [73-103]	0.74
**MAP, mmHg**	71 [66-76]	69 [66-103]	76 [68-82]	<0.01
**Temperature, °C**	37.2 [36.9-37.6]	37.2 [37.0-37.6]	37.1 [36.9-37.4]	0.82
**Mechanical ventilation, n (%)**	36 (90)	23 (92)	13 (87)	0.62
**FiO_2, %**	55 [50-60]	55 [43-60]	55 [50-60]	0.58
**PEEP, cmH_2O**	8 [8-10]	8 [7-10]	10 [8-10]	0.33
**pH**	7.36 [7.35-7.39]	7.36 [7.34-7.41]	7.35 [7.35-7.38]	0.63
**PaO_2, mmHg**	91 [69-101]	86 [64-99]	98 [90-112]	<0.01
**$PaCO_2$, mmHg**	41 [38-44]	41 [39-45]	40 [38-43]	0.27
**Lactate, mEq/L**	1.9 [1.5-2.3]	2.1 [1.8-2.9]	1.5 [1.2-2.1]	0.02
**Hb, gr/dL**	9.1 [8.7-9.9]	9.2 [8.7-9.9]	8.9 [8.7-9.9]	0.69

**Cardio-active medications**				
- **Norepinephrine, n (%)**	38 (95)	23 (92)	15 (100)	0.52
- **Dobutamine, n (%)**	8 (10)	5 (20)	3 (20)	1.00
- **Epinephrine, n (%)**	7 (18)	3 (12)	4 (27)	0.39
**TRANSCRANIAL DOPPLER AND PUPILLOMETRY FINDINGS**				
**FVm, cm/sec**	55 [53-62]	56 [53-65]	54 [53-59]	0.30
**eCPP, mmHg**	58 [50-67]	64 [57-71]	48 [43-57]	<0.01
**eICP, mmHg**	9 [4-24]	5 [3-8]	26 [18-32]	<0.01
**Impaired CA, n (%)**	21 (53)	13 (52)	8 (53)	1.00
**NPi**	4.3 [3.6-4.6]	4.5 [4.4-4.6]	3.5 [3.5-3.7]	<0.01
**Constriction velocity, mm/sec**	1.7 [1.5-2.3]	1.7 [1.5-2.5]	1.6 [1.3-2.1]	0.13
**Dilation velocity, mm/sec**	1.7 [1.2-2.8]	2.1 [1.3-3.0]	1.5 [1.2-2.6]	0.53
**Dead at ICU discharge, n (%)**	7 (18)	3 (12)	4 (27)	0.39

APACHE II acute physiology and chronic health evaluation II, CAR cerebral autoregulation, CKD chronic kidney disease, COPD chronic obstructive pulmonary disease, eCPP estimated cerebral perfusion pressure, eICP estimated intracranial pressure, FiO2 fraction of inspired oxygen, FVm mean intracranial blood flow velocity, Hb haemoglobin, MAP mean systemic arterial pressure, NPi neurological pupil index, $PaCO_2$ arterial carbon dioxide partial pressure, PaO_2 arterial oxygen partial pressure, PEEP positive end-expiratory pressure.
